# Knee Dislocation with Ipsilateral Tibial Fracture Treated with an Intramedullary Locked Nail and Simultaneous Transtibial Tunnel Knee Ligament Reconstruction: A Case Report of Autografts and Limited Resources

**DOI:** 10.1055/s-0040-1716685

**Published:** 2020-09-28

**Authors:** Túlio Vinícius de Oliveira Campos, Marcelo Nacif Moraes, Marco Antônio Percope de Andrade, Robert C. Schenck, Simon T. Donell

**Affiliations:** 1 Departamento de Aparelho Locomotor, Universidade Federal de Minas Gerais, Belo Horizonte, Minas Gerais, Brazil; 2 Orthopaedic Surgery Department, Hospital Risoleta Tolentino Neves, Sociedade Brasileira de Cirurgia do Joelho, São Paulo, Brazil; 3 Knee Surgery Department, Hospital da Baleia, Fundação Benjamin Guimarães, Belo Horizonte, Minas Gerais, Brazil; 4 Department of Orthopaedic Surgery and Rehabiliation, University of New Mexico Health Science Center, Albuquerque, New Mexico; 5 Knee Surgery Department, Norwich Medical School, Norwich, United Kingdom

**Keywords:** knee dislocation, tibia shaft fracture, posterior cruciate ligament, intramedullary nailing

## Abstract

Knee dislocations associated with ipsilateral tibial shaft fracture represent one of the most challenging injuries in trauma surgery. This injury occurs in only 2% of all tibial fractures in several series. With the use of intramedullary nail (IMN) of the tibia, current practice paraments suggest that transtibial tunnels should be avoided and ligamentous knee surgery be delayed until healing of the shaft fracture occurs. We report a novel case which was successfully managed by delayed IMN and multiligamentous transtibial posterior cruciate ligament (PCL) and posterolateral corner (PLC) autograft reconstructions. A 27-year-old male sustained a Gustilo-Anderson grade IIIa tibial shaft fracture and a Schenck IIIL knee dislocation (KD3L) in the ipsilateral knee. At 2 weeks, the patient was then taken back to the operating theater to undergo definitive bone fixation and ipsilateral simultaneous knee ligamentous reconstruction. The knee was stabilized by open reconstruction of the PCL under fluoroscopic control using an ipsilateral quadriceps autograft fixed with metallic interference screws. The PLC was reconstructed with ipsilateral semitendinosus autograft harvested through a separate 1.5-cm standard anteromedial incision using the technique described by Stannard et al. After graft fixation, the 90 degree posterior and posterolateral drawer and 0 and 30 degrees varus stress tests were negative. After 12 months follow-up, the patient had no complaints regarding pain or instability. The tibial fracture had healed and no knee axis deviation could be noted. The patient had returned to recreational low demand activities and motorcycle riding. Treatment of a combined tibial shaft fracture with an ipsilateral knee dislocation may be satisfactorily accomplished with an IMN for the tibia and transtibial tunnel fixation for knee ligament reconstruction allowing for a single rehabilitation course and a shorter recovery without having to use a third stage for knee ligamentous reconstruction.

## Introduction

Knee dislocations represent one of the most challenging injuries to manage. Associated fractures comprising an ipsilateral tibial shaft fracture often make immediate ligament reconstruction challenging. 1 2 Wascher et al were the first to report that 50% of knee dislocations spontaneously reduce, making the ligamentous injury difficult to identify and, if missed, the concomitant vascular injury could lead to limb or at least significant function loss. 3 4 Delay in the diagnosis of all injuries, may lead to inadequate treatment. 5 6 The association of tibial diaphyseal fracture and a multiligamentous knee injury is uncommon occurring in only 2% of all tibial fractures. 1 2 Intramedullary nailing (IMN) is the gold standard to manage diaphyseal fractures and most authors report difficulties with transtibial tunnels when approaching both lesions simultaneously. As a result, ligamentous knee surgery would be delayed until bone has healed and nail removed. We report a case which was successfully managed by initial damage control strategies using external fixation bridging both the knee and the tibial fracture followed by delayed one step IMN and multiligamentous transtibial PCL and PLC autograft reconstructions.

## Case Report

**Fig. 1:**
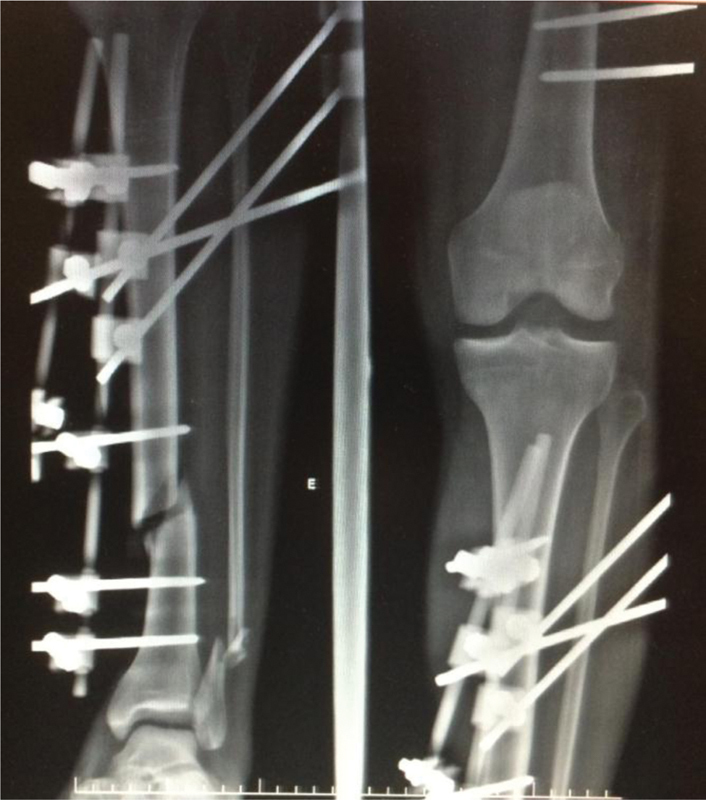
Plain radiograph of the left leg with the bridging external fixator applied to the tibial fracture and dislocated knee. Note the opening of the lateral compartment of the knee, suggesting PLC lesion. PLC, posterolateral corner.

A 27-year-old male was admitted to the Hospital Risoleta Tolentino Neves as an emergency victim following a high-speed motorcycle crash. He sustained a Gustilo-Anderson grade IIIa tibial shaft fracture and a Schenck KD3L in the ipsilateral knee. 3 7 8 Standard *Advanced Trauma Life Support* protocol and resuscitation management was applied and the patient was deemed stable for the operating room. Clinical examination revealed symmetrical pulses, soft compartments, and intact motor and sensory examinations. Doppler examination revealed normal flow to the affected extremity. The patient was taken to the operating room where examination of his knee under general anesthesia showed a grade III posterior drawer, a grade III anterior drawer, grade II Lachman, and positive varus stress examination opening at both 0 and 30 degrees. After debridement of the tibial wound a spanning external fixator including the knee joint was applied as shown in Fig. 1 . Clinical examination suggested complete tearing of the anterior cruciate ligament (ACL), PCL, and the PLC confirmed by subsequent magnetic resonance imaging.

**Fig. 2:**
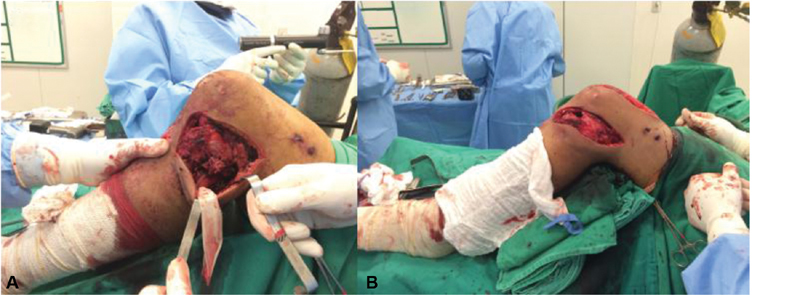
Intraoperative photograph showing a lateral view of the left leg. ( **A** ) The posterior sag of the knee can be seen; the common peroneal nerve is shown protected by a Penrose drain. The intramedullary nail was inserted through the most anterior portion of the incision. ( **B** ) Post-reconstruction view showing correction of the posterior sag.

The patient had an uneventful postoperative course. At 2 weeks the limb had ceased to be swollen and his open wounds had healed; the patient was then taken back to the operating theater to undergo definitive bone fixation and ipsilateral simultaneous knee ligamentous reconstruction. Afterward, the patient received general anesthesia and was positioned supine. A lateral curvilinear incision allowed both introduction of an intramedullary locked nail (Universal nail—Synthes) and one-stage reconstruction of the knee ligaments. The fibula was held reduced using a 2-mm intramedullary wire inserted retrograde through a stab incision. This helped define the tibial rotation and length before insertion of the tibial nail. The knee was stabilized by open reconstruction of the PCL under fluoroscopic control using an ipsilateral quadriceps autograft fixed by metallic interference screws. The PLC was reconstructed with ipsilateral semitendinosus autograft harvested through a separate 1.5-cm standard anteromedial incision using the technique described by Stannard et al 9 ( Fig. 2 )

After graft fixation, the posterior and posterolateral drawer at 90 degree and varus stress tests at 0 and 30 degrees were negative. The surgical incision was closed in layers and the knee immobilized by an extension splint. Patient was discharged home 3 days later with nonweight bearing crutches. At 3 weeks follow-up, an *Enterobacter cloacae* superficial infection was diagnosed. The wound was debrided and a 35 day course of oral flucloxacillin was prescribed. No further problems were noted.

The rehabilitation protocol comprised isometric quadriceps contraction exercises, prone supervised knee mobilization beginning on the first postoperative day, an extension brace nonweight bearing for 6 weeks followed by partial weight-bearing for further 6 weeks. Active range-of-motion exercises were allowed at week 8, and full weight bearing and closed-chain exercises at week 12.

**Fig. 3:**
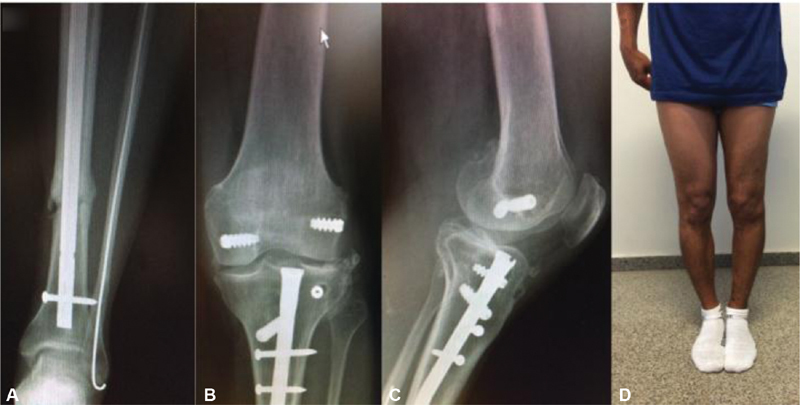
Follow-up images at 1 year with plain radiographs of the left leg ( **A–C** ) and a clinical photograph ( **D** ). ( **A** ) The tibial fracture healed. ( **B, C** ) AP and lateral view 1 year after surgical treatment. There is no joint line subluxation or opening of the knee joint. Degenerative changes are noted at the lateral compartment. ( **D** ) The left leg alignment matches the right. AP, anteroposterior.

After 12 months follow-up, the patient had no complaints regarding pain or instability. On clinical examination, there were no signs of knee malalignment; the knee range of motion was full and symmetrical with the contralateral leg, and the varus stress, posterior, and posterolateral drawer were negative ( Fig. 3 ). On follow-up X-rays, tibial fracture had healed with no axis deviation. The patient started recreational low demand activities and motorcycle riding. At final consultation, ACL reconstruction was not requested by the patient. The International Knee Documentation Committee Subjective Knee Form (IKDC) for the patient at 1-year follow-up was 55/87 which is 63.2%.

## Discussion

This is the first report of the use of an IMN to fix a tibia shaft fracture with PCL reconstruction through transtibial tunnels for a concomitant ipsilateral knee dislocation. Aydin et al 10 reported using external fixation for the tibial shaft fracture and conservative treatment of the associated knee dislocation. Huang et al 11 described two cases of isolated PCL rupture and tibia shaft fracture. The first patient was managed by open reduction and internal fixation using plates and screws for the tibia and PCL reconstruction, the second one had the tibia fracture approached by IMN and the PCL treated conservatively. Chahal et al 12 described a tibial shaft fracture associated with ACL, PCL, and medial collateral ligament injuries treated by IMN and tibial inlay reconstruction for the PCL. Gupta et al 13 treated a tibial shaft fracture associated with ACL, PCL, CPL, and popliteal artery injury with fixation of the fracture with plate and screws, open reconstruction of the ACL and PCL, and open repair of the PLC.

The concern that using an IMN for tibial shaft fracture fixation could jeopardize transtibial tunnel path was bypassed by careful planning of transtibial PCL tunnel position. The authors did consider utilizing a short tibial nail but a standard length was chosen as it allows nail removal in a more straightforward manner. The acute fracture fixation and knee ligamentous stabilization does carry a higher risk of stiffness, wound complications, and compartment syndrome. As a result, we chose to perform a staged approach, which included external fixation followed by definitive treatment. Preoperative patient education, limb edema regression, and articular capsule healing were responsible for the good clinical outcome. The advantage of using the IMN and ligament reconstruction simultaneously is that it allowed for a single rehabilitation process and a more predictable knee stability outcome by the early PLC reconstruction. Also, patient did not suffer knee arthrofibrosis as a consequence of preoperative patient education and early aggressive physical therapy range of motion exercises. Simultaneous fracture fixation and ligamentous reconstruction allowed for a satisfactory outcome from a potentially devastating injury.

Thiagarajan et al evaluated 50 consecutive patients with open tibial fracture. They reported that although the incidence of at least one ligament injury was 36%, only 22% were diagnosed in the first assessment. As a result, the authors recommended that all patients with severe tibial fracture had their knees examined focusing on multiligamentous injury diagnosis. 2 The case presented, confirms the relationship between tibial shaft fracture and knee ligament fracture.

## Conclusion

Treatment of a combined tibial shaft fracture and ipsilateral knee dislocation may be accomplished with an IMN for the tibia and transtibial tunnel fixation for knee ligament reconstruction when the traumatic inflammatory response to the soft tissues has settled, allowing for a single rehabilitation course and a shorter recovery without having to use a third stage for knee ligamentous reconstruction. Patient selection and preoperative education was also a factor in avoiding complications.
